# Conversion of rainforest to oil palm and rubber plantations alters energy channels in soil food webs

**DOI:** 10.1002/ece3.5449

**Published:** 2019-07-15

**Authors:** Winda Ika Susanti, Melanie M. Pollierer, Rahayu Widyastuti, Stefan Scheu, Anton Potapov

**Affiliations:** ^1^ J.F. Blumenbach Institute of Zoology and Anthropology University of Göttingen Goettingen Germany; ^2^ Department of Soil Sciences and Land Resources Institut Pertanian Bogor (IPB) Bogor Indonesia; ^3^ Centre of Biodiversity and Sustainable Land Use Göttingen Germany; ^4^ A.N. Severtsov Institute of Ecology and Evolution Russian Academy of Sciences Moscow Russia

**Keywords:** biomarker, fatty acids, land‐use change, macrofauna, mesofauna, soil fauna

## Abstract

In the last decades, lowland tropical rainforest has been converted in large into plantation systems. Despite the evident changes above ground, the effect of rainforest conversion on the channeling of energy in soil food webs was not studied. Here, we investigated community‐level neutral lipid fatty acid profiles in dominant soil fauna to track energy channels in rainforest, rubber, and oil palm plantations in Sumatra, Indonesia. Abundant macrofauna including Araneae, Chilopoda, and Diplopoda contained high amounts of plant and fungal biomarker fatty acids (FAs). Lumbricina had the lowest amount of plant, but the highest amount of animal‐synthesized C20 polyunsaturated FAs as compared to other soil taxa. Mesofauna detritivores (Collembola and Oribatida) contained high amounts of algal biomarker FAs. The differences in FA profiles between taxa were evident if data were analyzed across land‐use systems, suggesting that soil fauna of different size (macro‐ and mesofauna) are associated with different energy channels. Despite that, rainforest conversion changed the biomarker FA composition of soil fauna at the community level. Conversion of rainforest into oil palm plantations enhanced the plant energy channel in soil food webs and reduced the bacterial energy channel; conversion into rubber plantations reduced the AMF‐based energy channel. The changes in energy distribution within soil food webs may have significant implications for the functioning of tropical ecosystems and their response to environmental changes. At present, these responses are hard to predict considering the poor knowledge on structure and functioning of tropical soil food webs.

## 1 INTRODUCTION

Indonesia is a hotspot of biodiversity on Earth and at the same time a top world producer of agricultural products, such as oil palm (Fitzherbert et al., 2008; Koh & Ghazoul, 2010) and rubber (Marimin, Darmawan, Machfud, Putra, & Wiguna, 2014). Extension of agriculture in Indonesia was associated with deforestation which increased strongly in the last 20 years and this is predicted to continue (Gatto, Wollni, & Qaim, 2015; Koh & Ghazoul, 2010). Since the 1980s, after the transmigration program, large parts of rainforest in Jambi province, Sumatra, have been converted to oil palm (16% of total area) and rubber plantations (12%; Gatto et al., 2015). Thus, Sumatra represents an ideal model region to investigate the effect of rainforest conversion on biodiversity and ecosystem functioning at local and regional scale in Southeast Asia (Clough et al., 2016; Drescher et al., 2016).

Conversion of tropical rainforests into plantation systems is associated with changes in ecological niches of species and ultimately with the loss of species, and thereby with changes in ecosystem functioning (Barnes et al., 2014; Clough et al., 2016; Fitzherbert et al., 2008; Gilbert, 2012). Rainforest conversion strongly affects environmental processes, including soil organic carbon pools and soil erosion (Guillaume, Damris, & Kuzyakov, 2015), primary production of trees (Kotowska, Leuschner, Triadiati, Meriem, & Hertel, 2015), carbon dioxide and methane fluxes (Hassler et al., 2015), as well as nitrogen cycling and soil fertility (Allen, Corre, Tjoa, & Veldkamp, 2015). However, the effect of conversion of rainforest into plantations on belowground organisms is less well studied. It has been shown that rainforest conversion strongly affects biomass, vitality, and mycorrhizal colonization of roots (Sahner et al., 2015), biodiversity and abundance of microorganisms (Krashevska, Klarner, Widyastuti, Maraun, & Scheu, 2015; Schneider et al., 2015) and trophic‐guild composition of litter‐dwelling fauna (Barnes et al., 2014; Klarner et al., 2017). However, the effect of rainforest conversion on food resources of soil fauna and the relative importance of energy channels of soil food webs (Moore et al., 2004) is little understood. This is unfortunate, as the way energy is channeled through soil food webs is a major determinant of their stability (Ruiter, Neutel, & Moore, 1995; Moore & De Ruiter, 2012).

Rainforest conversion is likely to alter the flux of energy through soil food webs. For instance, nutrient inputs, changes in soil pH, as well as physical disturbances caused by management practices may shift fungal‐ and bacterial‐based energy channels in soils (Rousk et al., 2010). In temperate regions, more fertile and productive ecosystems foster the bacterial‐based energy channel, while less fertile ecosystems foster the fungal‐based energy channel (Wardle, 2004). Previous studies showed that conversion of rainforest into oil palm plantations results in a decreased amount of specific bacterial biomarker PLFAs in the litter (Krashevska et al., 2015) and causes a diet shift of generalist predators toward more herbivore prey (Klarner et al., 2017). However, the resulting changes in energy channels of the entire soil food web have not been explored. Soil organisms interact in complex ways in soil with their activity regulating ecosystem processes and delivering ecosystem services (Lavelle et al., 2006). Food web models allow calculating energy and nutrient fluxes based on consumer—resource interactions and the way how energy and nutrients are channeled through soil microbial and animal communities (De Ruiter, Van Veen, Moore, Brussaard, & Hunt, 1993). This channeling has been shown to be altered significantly with land‐use intensification (Wagg, Bender, Widmer, & Heijden, 2014). Building on these knowledge we explored changes in the channeling of energy through soil food webs after conversion of rainforest into plantation systems of rubber and oil palm.

Lipids play a vital role in animals both as storage compounds, for providing energy (neutral lipid fatty acids, NLFAs) and as structural component of cell membranes (phospholipid fatty acids, PLFAs; Ruess, Häggblom, García Zapata, & Dighton, 2002). Biomarker FAs are synthesized only by microorganisms and plants and transferred through food chains without modification, allowing to infer links between basal resources and higher order consumers (Chamberlain, Bull, Black, Ineson, & Evershed, 2005; Ruess & Chamberlain, 2010). For soil food web, it has been shown that fatty acids (FAs) are transferred from microorganisms to microbivorous invertebrates to higher order consumers (Chamberlain et al., 2005; Ruess et al., 2002; Ruess, Schütz, et al., 2005; Ruess, Tiunov, et al., 2005) as well as from microorganisms and plants to detritivores and their predators (Pollierer, Scheu, & Haubert, 2010). Thus, lipid profiles of consumers may serve as tool for soil food web diagnostics (Kühn et al., 2018). To‐date, however, virtually no studies investigated lipid profiles of soil fauna including both meso‐ and macrofauna in the same community.

Here, we used lipid profiles of soil fauna to track how energy channels in soil food webs are changing due to rainforest conversion into two major agricultural systems, that is, rubber and oil palm plantations, in Sumatra, Indonesia. We hypothesized that (a) different groups of meso‐ and macrofauna are linked to different basal resources with mesofauna detritivores, that is, Collembola and Oribatida, being closely associated with the fungal energy channel, and macrofauna detritivores such as Lumbricina and Diplopoda being more closely associated with the bacterial and plant energy channel. Further, based on results of previous studies (Klarner et al., 2017; Krashevska et al., 2015), which indicated reduced feeding on bacteria and increased feeding on plants with conversion of rainforest into oil palm plantations, we hypothesized (b) that rainforest conversion strengthens the plant‐based energy channel and reduces the bacterial energy channel in soil food webs of plantation systems.

## 2 MATERIAL AND METHODS

### 2.1 Study sites

Soil and litter samples were taken in lowland rainforest, rubber (*Hevea brasiliansis*) plantations and oil palm (*Elaeis guineensis*) plantations, located in Jambi province, southwest Sumatra, Indonesia. Jambi province stretches from the Barisan mountain range in the west across extensive lowlands toward the southern Malacca Strait in the east. The climate is tropical and humid with rainy seasons from March to December, and a dryer period during July and August (Drescher et al., 2016). Study sites were located at similar altitude varying between 50 and 100 m a.s.l. in Harapan landscape; each system was replicated four times (see Drescher et al., 2016 for more details). Rainforest sites used as reference comprised secondary rainforest close to natural condition that underwent selective logging some 20–30 years ago. Rubber and oil palm plantations were intensively managed monocultures of an average age of 6–16 and 8–15 years, respectively (Drescher et al., 2016). Soils at the study sites were loam acrisols of low fertility (Allen et al., 2015).

Management practices in these smallholder monoculture plantations are described in detail by Allen et al. (2015). In the loam acrisol soil, oil palm plantations were established after clearing and burning the previous jungle rubber whereas the rubber plantations were established from previously logged forest. Oil palms are fertilized once in the rainy season and once in the dry season. The most commonly used fertilizers are NPK complete fertilizer (i.e., Phonska and Mahkota), potassium chloride (KCl) and urea (CO(NH_2_)_2_). Both manual and chemical weeding took place throughout the year at the rubber and oil palm plantations. The most commonly used herbicides were Gramoxone and Roundup; these were applied at an average rate of 2–5 L herbicide ha^‐1^ year^‐1^ (Allen et al., 2015; Clough et al., 2016; Kotowska et al., 2015).

The description of major soil properties refers to the study by Krashevska et al. (2015) on the same field sites. Soil water content decreased between transformation systems in the order rainforest (39.94 ± 7.46%), rubber (38.22 ± 5.85%), and oil palm plantation (30.29 ± 6.47%). Soil pH was higher in converted systems and decreased in the order oil palm (4.97 ± 0.34), rubber (4.44 ± 0.11), and rainforest (3.95 ± 0.17). Total soil C concentration was higher in rainforest (4.25 ± 1.26%), but was similar in rubber (2.35 ± 0.73%) and oil palm plantation (2.57 ± 1.05%). Similarly, total soil N concentration was higher in rainforest (0.28 ± 0.06%), but was similar in rubber (0.19 ± 0.05%) and oil palm plantation (0.18 ± 0.04%). Soil C‐to‐N ratio was similar in rainforest (14.95 ± 1.56) and oil palm (14.31 ± 4.50), but lower in rubber plantation (11.64 ± 1.23).

### 2.2 Sampling

Samples were taken in September 2017 from three 5 × 5 m subplots within 50 × 50 m plots with a minimum distance of 200 m between plots established at each study site (Drescher et al., 2016). Each sample measured 16 × 16 cm and included the litter layer and underlying top soil to a depth of 5 cm. Since only few soil fauna were collected in oil palm plantation we performed an additional nonquantitative sampling of litter and soil in this system. Large soil fauna (>2 mm in body length; macrofauna) were collected by hand after sieving the litter through 2 cm mesh; small soil fauna (<2 mm; mesofauna) were extracted by heat (Kempson, Lloyd, & Gheraldi, 1963) during three days in water. Every day the extracted mesofauna species were picked manually from the water surface and identified to a family/order level under a dissecting microscope. To track the community‐level changes, fauna were collected nonselectively and then groups, dominated by biomass, were analyzed. The collection comprised six key groups: Collembola (families Entomobryidae and Paronellidae, and order Symphypleona), Oribatida (families Galumnidae, Otocepheidae, Galumnellidae, Scheloribatidae, Haplozetidae, Zetordestidae, and Lohmanniidae), Diplopoda (families Polydesmidae and Julidae), Araneae, Lumbricina, and Chilopoda (families Geophilomorpha and Scolopendromorpha; see Table S1 for the full list of taxa analyzed). To get enough material for lipid analysis, small fauna from different subplots and layers were bulked (in few cases, animals from two plots within the same system were combined); one sample consisted of 1–54 specimens of 0.5–2 mg fresh weight. After identification, animals were immediately transferred into pure methanol and stored at −20°C before fatty acid extraction (Zieger & Scheu, 2018). In total, 86 samples of soil fauna were analyzed. Data on fatty acid composition of all samples are available from Dryad Digital Repository (https://doi.org/10.5061/dryad.d6n77r3).

### 2.3 Analysis of fatty acids and biomarker assignment

NLFAs were extracted as described in Ruess, Schütz, et al. (2005)), Ruess, Schütz, et al. (2005)). In brief, animals were placed in 5 ml single phase extraction solvent (chloroform, methanol, 0.05 M phosphate buffer at a ratio of 1:2:0.8; pH 7.4) and NLFAs were extracted overnight on a shaker. The solvent was then transferred to new tubes, and the extraction was repeated by shaking for 1–2 hr with an additional 2.5 ml of extraction solvent. Extraction solvents of both steps were combined, 0.8 ml of distilled water and 0.8 ml of CHCl_3_ were added, and samples were centrifuged at 7**°**C in a multi centrifuge (402,48 *g*) for 5 min. Then, samples were allowed to stand to separate phases. The top two phases were removed and the chloroform fraction was transferred to a silica gel (SiOH) column (0.5 g, mesh size 100–200 µm). Lipids were eluted with 5 ml of chloroform, and the solvent was reduced by evaporation in a vacuum centrifuge. NLFAs were saponified and methylated following the procedure given for the Sherlock Microbial Identification System (MIDI Inc., Newark, Del). For saponification a solution of sodium hydroxide/methanol was used and the samples incubated at 100**°**C for 30 min followed by acid methanolysis in HCl‐methanol at 80**°**C for 10 min. The resulting fatty acid methyl esters (FAMEs) were stored at −20**°** until analysis.

FAMEs were transferred into vials, capped and analyzed by gas chromatography (Clarus 500, Perkin Elmer, Waltham, USA). Helium was used as carrier gas and NLFAs were identified by a flame ionization detector (capillary column: 30 m × 0.32 mm i.d., 0.25 mm film thickness; PE‐5, Perkin Elmer). The temperature program started with a temperature of 60°C (held for 1 min) and increased by 30**°**C per min to 160°C followed by 3°C per min to 260°C. The injection temperature was 250°C and helium was used as carrier gas. On the basis of retention time and comparison with standard mixtures, 37 different FAMEs ranging from C11 to C24 and 26 different BAMEs ranging from C11 to C20 were identified. Hexadecadienoic acid methyl ester and methyl‐hexadecatrienoate were used as algal biomarkers (SigmaeAldrich, St. Louis, USA).

Biomarker FAs for different taxa were assigned according to published studies (Table 1). To illustrate differences in proportions of biomarker FAs between taxa and systems, we used an array of biomarker FA‐based indices: Arbuscular mycorrhizal fungi (AMF) biomarker (16:1ω5), sum of plant biomarkers (18:3ω6,9,12; 18:1ω9; 22:0 and 24:0), fungal biomarker (18:2ω6,9), sum of algae biomarkers (16:2ω6,9 and 16:3ω6,9,12), sum of animal‐synthesized PUFAs (20:4ω6,9,12,15 and 20:5ω3,6,9,12,15), nonspecific bacteria/AM fungi biomarker (vaccenic type FAs 16:1ω7 and 18:1ω7), specific bacterial biomarkers (sum of all Gram + bacteria biomarkers i15:0, a15:0, i16:0, i17:0, and Gram‐ bacteria biomarkers cy17:0, cy 19:0, 2‐OH 12:0, 3‐OH 12:0, 2‐OH 14:0, 3‐OH 14:0), ratio between fungal biomarker and plant biomarkers, and ratio between fungal biomarker and specific bacterial biomarker FAs.

**Table 1:** List of fatty acids (FAs) used as biomarkers

FA	Resource	References
16:1ω5	Arbuscular mycorrhizal fungi (AMF)	Madan, Pankhurst, Hawke, and Smith (2002), Olsson (1999)
16:2ω6,9	Green algae	Buse, Ruess, and Filser (2013)
16:3ω3,6,9	Green algae	Buse et al. (2013)
20:4ω6,9,12,15	Animal‐synthesized^a^	Chamberlain et al. (2005), Chen, Ferris, Scow, and Graham (2001)
20:5ω3,6,9,12,15	Animal‐synthesized^a^	Chamberlain et al., 2005
18:2ω6,9	Fungi	Frostegard and Baath (1996), Haubert, Häggblom, Langel, Scheu, and Ruess (2006), Ruess and Chamberlain (2010), Ruess, Schütz, et al. (2005)); Ruess, Tiunov, et al. (2005))
16:1ω7	General/widespread bacteria	Haubert et al. (2006), Ruess and Chamberlain (2010), Ruess, Schütz, et al. (2005)), Ruess, Tiunov, et al. (2005)), Welch (1991), Zelles (1999)
18:1ω7	General/widespread bacteria	Welch (1991), Zelles (1999)
i15:0	Gram + bacteria	Haubert et al. (2006), Zelles (1999)
a15:0	Gram + bacteria	Haubert et al. (2006), Zelles (1999)
i16:0	Gram + bacteria	Haubert et al. (2006), Zelles (1999)
i17:0	Gram + bacteria	Haubert et al. (2006), Zelles (1999)
2‐OH 12:0	Gram − bacteria	Lee, Chan, Fang, and Lau (2004), Wakeham, Pease, and Benner (2003)
3‐OH 12:0	Gram − bacteria	Lee et al. (2004), Wakeham et al. (2003)
2‐OH 14:0	Gram − bacteria	Lee et al. (2004), Wakeham et al. (2003)
3‐OH 14:0	Gram − bacteria	Lee et al. (2004), Wakeham et al. (2003)
cy17:0	Gram − bacteria	Zelles (1999)
2‐OH 16:0	Gram − bacteria	Lee et al. (2004), Wakeham et al. (2003)
cy19:0	Gram − bacteria	Zelles (1999)
18:3ω6,9,12	Plants	Millar, Smith, and Kunst (2000), Ruess et al. (2007)
18:1ω9	Plants	Harwood and Russell (1984), Ruess, Schütz, et al. (2005)), Ruess, Tiunov, et al. (2005)), Ruess et al. (2007)
22:0	Plants	Ruess et al. (2007), Zelles (1999)
24:0	Plants	Ruess et al. (2007), Zelles (1999)

a Animal‐synthesized C20 polyunsaturated FAs cannot be used as biomarkers for animal consumption.

### 2.4 Statistical analysis

Statistical computations were done in R version 3.4.0 (R Core Team, 2017) with R Studio interface (R studio Inc.). To normalize the data across different taxa and sites, we analyzed proportions of total (%) of individual NLFAs instead of the initial peak areas. We first calculated mean proportions for each NLFA for each group on each plot. In cases where more than one sample of the same group was analyzed per plot, we calculated mean proportions across these samples. We used the mean values for each taxon on each plot as replicates in statistical analyses (Table S2). Proportional data were arcsine‐ and logit‐transformed. After visual inspection of distributions for individual NLFAs, arcsine transformation was chosen for further statistical analyses, as it was more close to normal.

To analyze the effect of taxonomic group on individual biomarker FAs and NLFA indices in soil fauna, we used a subset of dominating groups found on at least four plots across at least two ecosystems, which were classified as macrofauna predators (Araneae and Chilopoda), macrofauna detritivores (Diplopoda and Lumbricina), or mesofauna detritivores (Collembola and Oribatida). Since different groups dominated different ecosystems and plots, we were not able to run a reliable full‐design model with both taxonomic group and ecosystem type as fixed factors. Thus, we built a set of linear mixed‐effects models with individual biomarker FAs as response variables and ecosystem (rainforest, rubber or oil palm) as random effect using the *lme4* package (Bates, Mächler, Bolker, & Walker, 2015). Significance of the overall effect was evaluated using the *lmerTest* package (Kuznetsova, Brockhoff, & Christensen, 2016); significant differences between groups were tested using general linear hypothesis Tukey test in the *multcomp* package (Hothorn, Bretz, & Westfall, 2008). *Vice versa*, to analyze the effect of ecosystem on individual biomarker FAs in soil fauna, we used a set of linear mixed‐effects models with taxonomic group as random effect. Taxonomic group was used as random effect since we were interested in shifts of community food resources irrespective of community taxonomic composition. In addition, we analyzed shifts in NLFA composition between ecosystems using linear discriminant analysis (LDA) and multivariate analysis of variance (MANOVA) based on seven NLFA indices in *vegan* and *MASS* packages. Data are presented as means ± 1 *SD*; *p* < .05 was used as the level of significance.

## 3 RESULTS

### 3.1 Food resources of different groups of soil fauna

**Figure 1:**
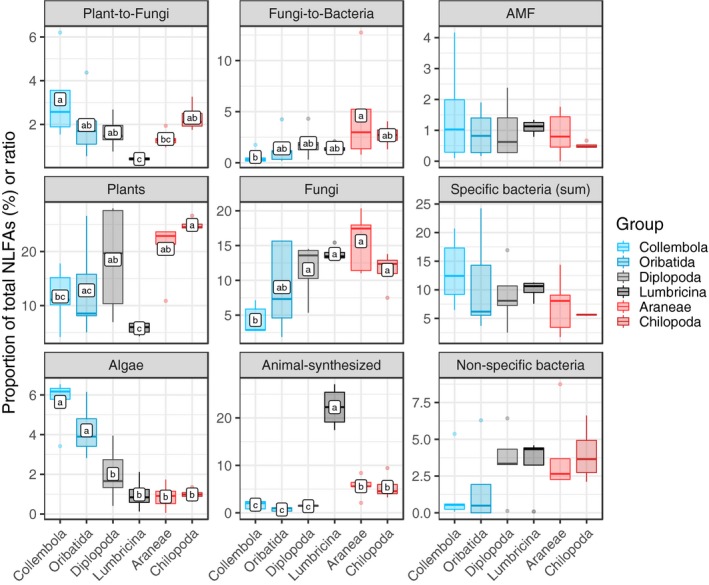
Biomarker fatty acid‐based indices in dominating soil fauna across the studied ecosystems. All indices are given as proportion of total biomarker fatty acids (FAs) except for plant‐to‐fungi and fungi‐to‐bacteria biomarker FA ratios (see Methods). Taxa include two groups of detritivore mesofauna (Collembola and Oribatida, blue), two groups of detritivore macrofauna (Diplopoda and Lumbricina, gray) and two predatory groups (Araneae and Chilopoda, red). Individual biomarker FAs with the same (or absent) letter are not significantly different according to Tukey's honestly significant difference test (*p* > .05)

Twenty‐three biomarker FAs were grouped into seven categories representing different basal resources and two indices were calculated: plant‐to‐fungi and fungi‐to‐bacteria (Table 1; Figure 1). In general, oleic acid (18:1ω9) as relative biomarker FA for plants and linoleic acid (18:2ω6,9) as relative biomarker for fungi were the most common fatty acids in soil fauna across ecosystems and taxa (Table S3). Groups of macrofauna (Araneae, Chilopoda, and Diplopoda) contained about twice higher proportions of fungal and plant biomarker FAs (Figure 1) as compared to mesofauna groups (Collembola and Oribatida). Mesofauna detritivores (Collembola and Oribatida) contained four times higher proportions of algal biomarker FAs as compared to macrofauna groups. Collembola contained the highest proportion of algal biomarker, and the lowest proportion of fungal biomarker FAs across groups. This resulted in the highest ratio of plant‐to‐fungi and the lowest ratio of fungi‐to‐bacteria in Collembola. The concentration of Gram‐ bacteria biomarker FAs varied among groups, but tended to be higher in mesofauna than macrofauna; Oribatida contained the highest proportion of the Gram‐ bacteria biomarker FA 2‐OH 14:0 and Collembola contained the highest proportion of the Gram‐ bacteria biomarker FA cy17:0 (Table S3). Lumbricina contained the lowest proportion of plant biomarker FAs, but the highest proportion of fungal biomarker FAs and had by far the lowest ratio of plant‐to‐fungi biomarker FAs. Animal‐synthesized (C20 polyunsaturated) FAs were four times higher in predators than in most detritivores except for Lumbricina that contained four times higher proportion of animal‐synthesized FAs than both predatory groups (Araneae and Chilopoda). The proportion of the AMF and nonspecific bacteria biomarker FAs did not differ significantly between taxonomic groups, but the latter were about twice lower in mesofauna than in macrofauna (Figure 1).

### 3.2 Effect of land‐use change on food resources of soil fauna

**Figure 2:**
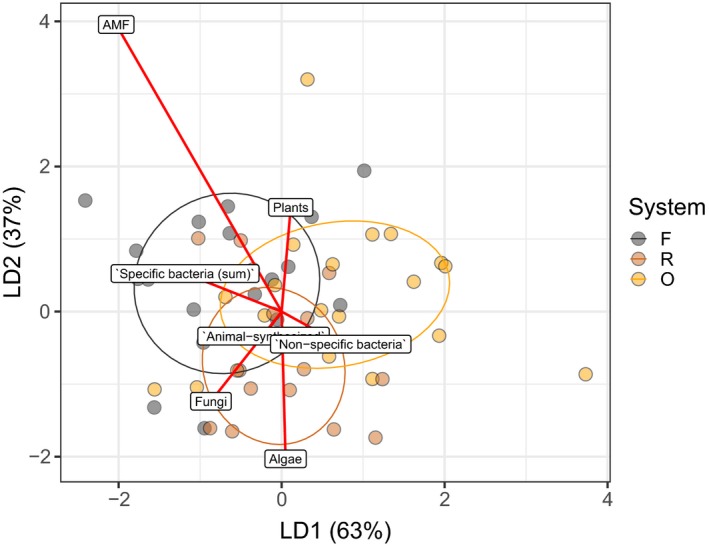
Linear discriminant analysis of biomarker fatty acid (FA) composition in soil fauna from rainforest, rubber and oil palm plantations. Each point represents a sample (an animal or a group of animals); all groups are plotted together, seven biomarker FA indices were included in the analysis (Table 1). Colors represent land‐use systems: F—rainforest (gray), R—rubber plantation (brown), O—oil palm plantation (orange). Ellipses are drawn on 60% confidence limits

Land use affected the NLFA indices‐based profiles, if all groups of soil fauna were analyzed together (MANOVA: *F*_2,14_ = 1.82, *p* = .0469; Figure 2). The first axis of LDA, explaining 63% of distinction between land‐use systems, was related to specific and nonspecific bacterial biomarker FAs with the proportion of the former being higher in animals from rainforest and the latter being higher in animals from plantation systems (Table S4). AMF biomarker NLFA was positively associated with animal taxa in rainforest and negatively with animal taxa in plantation systems, especially in rubber. Plant and algae biomarker FAs were negatively intercorrelated, with the proportion of the former being higher in animals from rainforest and oil palm plantations, and the latter being higher in animals from rubber plantations. The proportion of the fungal biomarker FA was lower in animals from oil palm plantations. Animal‐synthesized FAs showed no clear trend (Figure 2).

**Figure 3:**
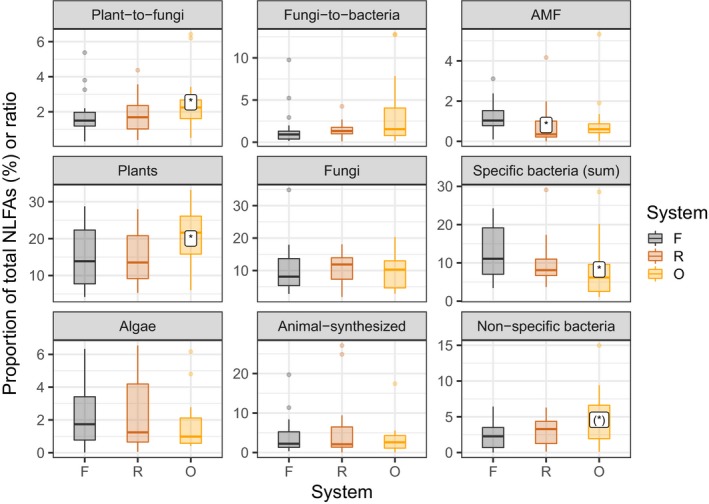
Biomarker fatty acid (FA)‐based indices in soil fauna from different land‐use systems. All indices are given as proportions of total biomarker FAs except for plant‐to‐fungi and bacteria‐to‐fungi FA ratios (see Methods). Data are bulked for soil fauna groups. Significant differences between plantation systems and forest (as the reference) are based on linear mixed effects models and indicated by asterisks (*p* < .05). Colors represent land‐use systems: F—rainforest (gray), R—rubber plantation (brown), O—oil palm plantation (orange)

**Figure 4:**
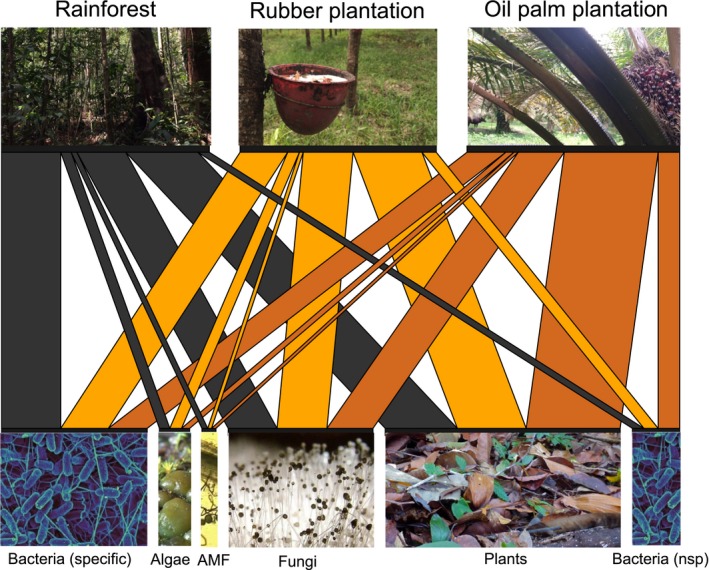
Channeling of energy from basal resources into consumers in rainforest, rubber and oil palm plantations as indicated by fatty acid analysis. The width of channels to respective land use systems reflects the relative importance of the channels as indicated by the mean proportion of biomarker neutral lipid fatty acids (NLFAs) of total NLFAs in soil fauna in the respective land‐use systems (data bulked across animal groups). The width of boxes/pictures of basal resources reflects the relative importance of these resources across land‐use systems; note that it ignores potential differences in production and assimilation rate of different NLFAs among producer and consumer groups. Colors represent land‐use systems: F—rainforest (gray), R—rubber plantation (brown), O—oil palm plantation (orange). Figure prepared using the R package *bipartite*

Overall, out of 23 biomarker FAs analyzed, seven differed significantly between land‐use systems (Table S5). Out of eight indices analyzed, five differed significantly between land‐use systems, that is, AMF, plant, specific bacteria (Gram + bacteria, Gram − bacteria), nonspecific bacteria and plant‐to‐fungi ratio (Figure 3). The proportion of the AMF biomarker NLFA 16:1ω5 was ca. 33% lower in soil fauna in rubber plantations as compared to soil fauna in rainforest. The proportion of the plant biomarker FA 18:3ω6,9 was 33% higher in soil fauna in oil palm plantations than in soil fauna in rainforest and rubber plantations. This resulted in the highest plant‐to‐fungi biomarker ratio in oil palm plantations. The proportion of specific bacteria biomarker FAs including those of gram+ (a15:0, i16:0, and i17:0) and gram− bacteria (cy17:0) was highest in soil fauna from rainforest and decreased gradually to rubber and oil palm plantations (in total, by about 35%). The opposite was true for the proportion of the nonspecific bacteria biomarker FA 16:1ω7 which was 70% higher in soil fauna in oil palm plantations than in soil fauna in other land‐use systems (Figure 4).

## 4 DISCUSSION

Conversion of rainforests into rubber and oil palm plantations alters energy channels in the soil food web as shown by different composition of biomarker FAs in soil fauna communities in different land‐use systems. Our results show a general increase in the plant energy channel and strong alterations in the bacterial energy channel in oil palm plantations, and a reduction of the AMF‐root‐based energy channel in rubber plantations as compared to rainforest. Despite the general shifts in the use of basal food resources, our results also showed that different trophic groups and size classes of soil fauna contain different specific biomarker FAs across land‐use systems and thus are linked to different basal resources. In most of the cases, the group identity had a larger effect on the FAs composition than land‐use change and thus this effect will be discussed in the first place.

### 4.1 Food resources of different groups of soil fauna

The plant biomarker 18:1ω9 and the fungal biomarker 18:2ω6,9 were the most common biomarker FAs in the majority of soil fauna groups, which is in line with other studies (Pollierer, Dyckmans, Scheu, & Haubert, 2012; Ruess et al., 2002). Soils contain high amounts of plant residues which are colonized by fungi and serve as basal resources/food for many groups of detritivore soil fauna. Via predator–prey interactions, these resources are transferred to higher trophic levels (Pollierer et al., 2010; Ruess & Chamberlain, 2010). Linoleic acid (18:2ω6,9) in NLFAs of soil fauna may also be derived from de novo synthesis with oleic acid 18:1ω9 as precursor via lipid metabolism (Menzel, Ngosong, & Ruess, 2017; Ruess et al., 2007).

Contrasting our first hypothesis, the proportion of the fungal biomarker FA was unexpectedly low in Collembola and Oribatida as compared to other soil fauna groups, although these detritivore mesofauna taxa are assumed to predominantly feed on fungi (Chahartaghi, Langel, Scheu, & Ruess, 2005; Klironomos & Kendrick, 1995; Lenoir, Persson, Bengtsson, Wallander, & Wirén, 2007; Scheu & Falca, 2000; Visser, Whittaker, & Parkinson, 1981). Previous studies from temperate forest ecosystems found indeed a high proportion of fungal biomarker FAs in these groups (Chamberlain et al., 2005; Chen, Sandmann, Schaefer, & Scheu, 2017; Haubert, Häggblom, Scheu, & Ruess, 2004; Ruess, Häggblom, Langel, & Scheu, 2004). Based on FA analysis, Ferlian, Klarner, Langeneckert, and Scheu (2015) reported plant, fungi, and bacteria as major resources for Collembola in a temperate ecosystem. Plant and fungal biomarker FAs represented 34% and 27% of total NLFAs in Collembola, respectively (Haubert et al., 2004), and up to 80% of total NLFAs in Oribatida (Pollierer et al., 2012). However, Ngosong, Raupp, Scheu, and Ruess (2009) reported that fungi were of minor importance for Collembola nutrition in an arable soil. In our study, plant and fungal biomarker FAs represented only 13% and 4% of total NLFAs in Collembola, respectively, and 15% and 9% in Oribatida, respectively. Rather than on fungi, NLFA analysis suggests that both Collembola and Oribatida predominantly feed on bacteria (about 12%–15% of their total NLFAs were represented by specific and nonspecific bacterial biomarker FAs). Further, our results indicate that in the studied tropical ecosystems both Collembola and Oribatida to some extent feed on algae, with on average algal biomarker FAs comprising 6% of total NLFAs in Collembola and 4% in Oribatida. Photoautotrophic algae and bacteria may represent a considerable portion of the diet of Collembola in various habitats (Potapov, Korotkevich, & Tiunov, 2018; Schmidt, Dyckmans, & Schrader, 2016). A high palatability of algae for Collembola and Oribatida was demonstrated in laboratory experiments (Brückner, Schuster, Smit, & Heethof, 2018; Buse, Ruess, & Filser, 2013, 2014; Scheu & Folger, 2004) and field stable isotope‐based studies from temperate forest ecosystems suggested that algae serve as food resource for certain species of Collembola and Oribatida (Schneider et al., 2004; Maraun et al., 2011; Potapov, Semenina, Korotkevich, Kuznetsova, & Tiunov, 2016). However, field data on algal biomarker FAs from temperate ecosystems are scarce since biomarkers for algae only were suggested recently (Buse, Ruess, & Filser, 2013, 2014). Overall, the results indicate that the diet of Collembola and Oribatida in tropical ecosystems predominantly consists of bacteria and to some extent on algae, contrasting temperate and boreal ecosystems where these animals predominantly feed on fungi and dead organic matter. This shift in diet may be related to low food quality of litter in tropical ecosystems (Hättenschwiler, Coq, Barantal, & Handa, 2011; Illig, Reinhard, Norton, & Scheu, 2005; Krashevska et al., 2018; Marian, Sandmann, Krashevska, Maraun, & Scheu, 2017, 2018). Further, low quality of litter associated with low availability of fungi as food might be responsible for the lack of primary decomposers (Illig et al., 2005) and low abundance of Collembola and Oribatida in tropical ecosystems (Marian, Sandmann, Krashevska, Maraun, & Scheu, 2018).

Biomarker FA composition of Diplopoda suggests that they predominantly feed on plant material and fungi, less on bacteria and only little on algae. This is in line with previous studies reporting that Diplopoda typically function as primary decomposers by feeding on plant litter (Bonkow, Scheu, & Schaefer, Scheu & Schaefer, 1998; Cárcamo, Abe, Prescott, Holl, & Chanway, 2000). Supporting this notion, Diplopoda readily feed on decomposing leaf litter of tropical trees in the laboratory (Ashwini & Sridhar, 2005). High ^13^C and low ^15^N enrichment indicates that litter‐dwelling Diplopoda likely assimilate plant material colonized by saprotrophic fungi (Potapov, Semenina, Kurakov, & Tiunov, 2013; Potapov, Tiunov, & Scheu, 2018; Semenyuk, Tiunov, & Golovatch, 2011). Similarly, high concentrations of plant biomarkers in Diplopoda of temperate systems (Ambarish & Sridhar, 2015; Rawlins, Bull, Poirier, Ineson, & Evershed, 2006) as in the present study suggest that litter‐dwelling Diplopoda occupy similar trophic niches in temperate and tropical ecosystems.

Among the soil fauna groups studied, Lumbricina contained the highest amount (on average 22%) of C20 carbon polyunsaturated FAs (C20 PUFAs; 20:4ω6,9,12,15 and 20:5ω3,6,9,12,15) which are assumed to be of animal origin (Albro, Schroeder, & Corbett, 1992; Sampedro, Jeannotte, & Whalen, 2006). As Lumbricina are detritivores that live in and feed on soil organic matter these FAs presumably are synthesized predominantly by the animals themselves as suggested earlier (Chamberlain et al., 2005; Petersen & Holmstrup, 2000) rather than originate from the diet. However, Sampedro et al. (2006) also found high concentrations of C20 PUFAs in the digestive tract of Lumbricina suggesting that at least in part they may also originate from the diet, possibly by feeding on protozoa and other soil microfauna (Aira, Monroy, & Dominguez, 2003; Bonkowski & Schaefer, 1997; Domínguez, Parmelee, & Edwards, 2003; Monroy, Aira, & Domínguez, 2008; Pokarzhevskii, 1997; Sampedro et al., 2006). Bacterial and fungal biomarker FAs were found to comprise up to 14%, whereas plant biomarker FAs only 6% of total NLFAs in Lumbricina. This also is in line with results of Sampedro et al. (2006) who showed that bacterial and fungal biomarker FAs are more abundant than plant biomarker FAs in Lumbricina suggesting that they little assimilate plant‐based resources. Studies on tropical Lumbricina (Lattaud, Zhang, Locati, Rouland, & Lavelle, 1997; Lavelle et al., 1987) also revealed that digestion system in association with soil microflora is very efficient and to digest soil organic matter through a mutualist Lumbricina microflora‐digestion system and the intestinal mucus was supposed to play a central role in the process of digestion. The similarity in lipid profiles and gut content of Lumbricina in our study and published data from temperate and tropical ecosystems suggests that Lumbricina occupy a similar trophic niche across biomes and function as trophic level omnivores feeding on detritus, microorganisms, and microfauna.

Araneae and Chilopoda are generalist predators which commonly feed on Collembola (Eitzinger, Rall, Traugott, & Scheu, 2018; Rusek, 1998; Voigtländer, 2011). Unexpectedly and contrasting our first hypothesis, the biomarker FA composition of both Araneae and Chilopoda was different from that of Collembola, and comprised high proportions of plant and fungal biomarker FAs. Since Araneae are generalist predators, they can consume prey from both the decomposer and herbivore system (Oelbermann, Langel, & Scheu, 2008; Wise, 2004, 2006). The similarly high concentrations of plant biomarker FAs in Araneae and Chilopoda suggest that, in contrast to boreal and temperate systems, these predators heavily rely on herbivorous prey in tropical systems. Conform to this finding, generalist predators have been shown to be able to regulate populations of pest species in tropical agricultural systems (Panizzi & Corrêa‐Ferreira, 1997; Sigsgaard, 2000). In addition, predators contained higher amounts of C20 PUFAs than decomposer animals, suggesting that in addition to synthesizing these FAs they also incorporate significant amounts from their diet which makes C20 PUFAs a potential trophic level biomarker (but there are some remarkable exceptions, such as Lumbricina).

### 4.2 Effect of land‐use change on food resources of soil fauna

Despite the majority of NLFAs in soil fauna in all studied land‐use systems originated from plants and fungi, the relative proportions of several NLFA biomarkers varied systematically among the systems. Due to the large spatial scale of the study and the limited number of replicates analyzed variability in NLFA, data were high. However, the fact that differences between land‐use systems were significant suggests that changes would even be more pronounced if more data would have been available. The high abundance of plant biomarker FAs and high plant‐to‐fungi NLFA ratio suggests that the soil fauna is closely linked to plant basal resources in oil palm plantations. Supporting this conclusion, based on changes in stable isotope ratios, Klarner et al. (2017) found generalist litter‐dwelling predators (Chilopoda) in oil palm plantations to more heavily rely on plant‐based food chains than in rainforest. Our results suggest that this not only applies to generalist predators but also to the entire soil animal community of oil palm plantations. Presumably, this is due to the well‐developed herb layer in oil palm plantations and occasional weeding increasing the input of high‐quality plant material to the belowground system. Overall, the results indicate that conversion of rainforest to oil palm plantations increases the plant‐based energy channel in soil food webs.

The AMF biomarker NLFA showed different trends in plant litter (Krashevska et al., 2015) and in soil fauna. Investigating the same study sites, Krashevska et al. (2015) found the AMF biomarker NLFA in litter to be lowest in rainforest litter and not to differ significantly between land‐use systems in soil, whereas we found the proportion of AMF biomarker NLFAs in soil fauna to be highest in rainforest. The Acrisol soils at our study sites are poor in nutrients (Allen, Corre, Kurniawan, Utami, & Veldkamp, 2016), potentially forcing AMF to exploit nutrients in litter. The AMF biomarker NLFA was low in fauna from rubber plantations indicating that soil fauna little feed on AMF in this land‐use system. Low AMF abundance in rubber plantations likely is related to high root phosphorus concentration (Bolan, 1991; Li, Smith, Holloway, Zhu, & Smith, 2006) and reduced energy flow from leaves to roots due to cutting the phloem for collecting rubber. Our results suggest that the root‐based energy channel in soil food webs of rubber plantations is reduced (Figure 4).

In line with the study of Krashevska et al. (2015), we found the abundance of nonspecific bacterial biomarker FAs to be highest in oil palm plantations while the abundance of specific bacterial biomarker FAs decreased. Based on metagenomics, Schneider et al. (2015) also found certain bacteria taxa to be more abundant in oil palm plantations as compared to rainforest. The increase likely is due to more beneficial environmental factors, in particular increased soil pH (Nacke et al., 2011; Rousk et al., 2010), base saturation (Schneider et al., 2015) and/or fertilization (Shen, Zhang, Di, & He, 2012). The increase in soil pH in plantation systems as compared to rainforest presumably is due to ashes from plant biomass burning (van Straaten et al., 2015) and liming (Allen et al., 2016; Krashevska et al., 2015). The more open canopy and improved light conditions in oil palm plantations as compared to rainforest may also favor photosynthetically active bacteria (Schneider et al., 2015). However, changes in nonspecific bacterial biomarker FAs may not be related to changes in bacterial community composition in a straightforward way. De novo synthesis of nonspecific bacterial biomarkers (vaccenic type FAs 16:1ω7 and 18:1ω7) was shown for fungi and protozoa (Devillard, McIntosh, Newbold, & Wallace, 2006; Maia, Chaudhary, Figueres, & Wallace, 2007; Or‐Rashid, AlZahal, & McBride, 2008; Or‐Rashid, Odongo, & McBride, 2007). Even some animals such as Collembola can synthesize these FAs (Pollierer et al., 2010). Olsson (1999) also reported that AMF can synthesize vaccenic type FA 16:1ω7. Although soil pH and base saturation is higher in rubber and oil palm plantations, rainforest has higher soil water content, soil C and N concentrations and amount of litter (Krashevska et al., 2015; van Straaten et al., 2015), resulting in more favorable conditions for bacteria and thereby strengthening the bacterial energy channel (Wardle et al., 2004; Figure 4). Conform to these findings, the absolute biomarker FAs for Gram+ (a15:0, i16:0, i17:0) and Gram‐ bacteria (cy17:0) in soil fauna significantly declined from rainforest to rubber to oil palm plantations (Table S5). This decline mirrors the decrease in biomarker PLFAs cy17:0 and i17:0 in litter of rubber and oil palm plantations as compared to rainforest (Krashevska et al., 2015), suggesting that soil meso‐ and macrofauna nonselectively feed on bacteria.

## 5 CONCLUSIONS

The results suggest that different high‐rank taxonomic groups are linked to different resources. Fungi appear to contribute only little to the nutrition of detritivore mesofauna taxa, in particular Collembola, contrasting temperate and boreal ecosystems. Further, algae are likely to play a more important role as food resource of mesofauna (Collembola and Oribatida) in tropical than temperate ecosystems. Litter‐dwelling macrofauna (Araneae, Chilopoda, and Diplopoda) heavily rely on fungal‐ and plant‐based resources, while soil‐dwelling Lumbricina mostly rely on microbial resources, but only little on plant‐based resources such as leaf litter. Differences between soil fauna groups were rather constant across land‐use systems, suggesting a consistent niche differentiation between different trophic groups and body size classes (meso‐ and macrofauna) in soil. The unexpected differences in trophic niches of some fauna groups between temperate and tropical ecosystems call for further studies to understand the driving factors for these differences.

Changes in energy channels with conversion of rainforest into plantation systems suggest that soil fauna communities respond in a flexible way to changes in the availability of food resources. Soil fauna in oil palm plantations predominantly rely on plant‐based resources. By contrast, the soil fauna community in rainforest heavily relies on the bacterial energy channel. The contribution of AMF in fueling soil food webs appears to be particularly low in rubber plantations, presumably due to alleviated limitation by phosphorus and by cutting the phloem for harvesting rubber. These alterations suggest that land‐use change alters food‐web structure and may disrupt interaction networks resulting in changes in ecosystem functioning and food‐web resilience that are hard to predict with the current limited knowledge on tropical belowground systems.

## CONFLICT OF INTEREST

None declared.

## AUTHOR CONTRIBUTIONS

AP designed the study and performed field sampling. WS analyzed fatty acid composition of fauna and wrote the manuscript. All authors contributed critically to drafts and gave their final approvement for publication.

## Data Availability

Data supporting the results were archived in the Dryad repository (https://doi.org/10.5061/dryad.d6n77r3) Susanti (2019).
