# Supplementary material for: Conversion of rainforest to oil palm and rubber plantations alters energy channels in soil food webs
Source: Ecol Evol. 2019 Jul 15;9(16):9027–39. doi: 10.1002/ece3.5449 (PMC6706186; doi:10.1002/ece3.5449)
Supplement: Supplementary file 1 [file ECE3-9-9027-s001.docx]

**Supplementary Table S1**. List of soil fauna used for NLFA analyzes.

| **No.** | **System** | **Plot_ID** | **Group** | **Subroup** | **Number of animals** | **Comments** |
| --- | --- | --- | --- | --- | --- | --- |
| 1 | Rainforest | HF1&3 | Araneae | Araneae | 8 | HF1 and HF3, small free-hunting Araneae |
| 2 | Rainforest | HF2 | Blattodea | Blattodea | 1 | Large size, wingless |
| 3 | Rainforest | HF1&3 | Diplura | Campodeidae | 5 | HF2 and HF3 |
| 4 | Rainforest | HF2 | Coleoptera | Carabidae - imago | 6 | Small size, brown |
| 5 | Rainforest | HF4 | Coleoptera | Carabidae - imago | 8 | Small size, brown |
| 6 | Rainforest | HF1&3 | Lumbricina | Clitellata | 1 |  |
| 7 | Rainforest | HF1&3 | Lumbricina | Clitellata | 2 | Medium size |
| 8 | Rainforest | HF1&3 | Lumbricina | Clitellata |  |  |
| 9 | Rainforest | HF1&3 | Coleoptera | Coleoptera - imago | 1 | cf. Agathidium |
| 10 | Rainforest | HF1&3 | Coleoptera | Coleoptera - larvae | 2 | HF1 and HF3 larvae cf. Elateridae |
| 11 | Rainforest | HF1&3 | Coleoptera | Coleoptera - larvae | 1 | larvae cf. Elateridae, 1 large |
| 12 | Rainforest | HF4 | Coleoptera | Coleoptera - larvae | 3 | larvae cf. Elateridae, 1 large, 2 small |
| 13 | Rainforest | HF2 | Chilopoda | Geophilomorpha | 1 | Large size |
| 14 | Rainforest | HF2 | Chilopoda | Geophilomorpha | 1 | Medium size |
| 15 | Rainforest | HF2 | Chilopoda | Geophilomorpha | 1 | Medium size |
| 16 | Rainforest | HF4 | Araneae | Hunting Araneae | 4 | Pale, medium size, free-hunting |
| 17 | Rainforest | HF4 | Isopoda | Isopoda | 1 | juvenile cf. Oniscidae |
| 18 | Rainforest | HF1&3 | Diplura | Japygidae | 25 | HF1 and HF3 |
| 19 | Rainforest | HF2 | Collembola | Lepidocyrtini | 13 | Sinella + Lepidocyrtus |
| 20 | Rainforest | HF4 | Collembola | Lepidocyrtini | 50 | Sinella + Lepidocyrtus |
| 21 | Rainforest | HF2 | Mesostigmata | Mesostigmata | 15 | Gamasina |
| 22 | Rainforest | HF4 | Opiliones | Opiliones | 2 | Small size, „mite-harvestman“ |
| 23 | Rainforest | HF2 | Oribatida | Oribatida | 23 | Galumnidae, Otocepheidae, Galumnellidae, Scheloribatidae |
| 24 | Rainforest | HF4 | Oribatida | Oribatida | 34 | Galumnidae, Haplozetidae, Galumnellidae, Zetordestidae |
| 25 | Rainforest | HF2 | Collembola | Paronellidae | 1 | 1 large |
| 26 | Rainforest | HF1&3 | Diplopoda | Polydesmidae | 1 | Medium size |
| 27 | Rainforest | HF2 | Diplopoda | Polydesmidae | 3 | Medium size |
| 28 | Rainforest | HF2 | Diplopoda | Polydesmidae | 1 | Medium size |
| 29 | Rainforest | HF2 | Pseudoscorpionida | Pseudoscorpionida | 16 |  |
| 30 | Rainforest | HF4 | Schizomida | Schizomida | 2 | 1 large, 1 small |
| 31 | Rainforest | HF1&3 | Symphyla | Symphyla | 54 | HF1 and HF3 |
| 32 | Rainforest | HF2 | Collembola | Symphypleona | 8 |  |
| 33 | Oil Palm | HO2 | Araneae | Araneae | 4 | 1 small + 1 mrdium brown white legs, 1 brown small, 1 black small white legs, free hunters |
| 34 | Oil Palm | HO1 | Blattodea | Blattodea | 1 | juvenile |
| 35 | Oil Palm | HO2 | Blattodea | Blattodea | 1 | juvenile |
| 36 | Oil Palm | HO3&4 | Blattodea | Blattodea | 1 | juvenile |
| 37 | Oil Palm | HO3&4 | Lumbricina | Clitellata | 1 |  |
| 38 | Oil Palm | HO1 | Dermaptera | Dermaptera | 2 | Medium, brown |
| 39 | Oil Palm | HO1 | Dermaptera | Dermaptera | 2 | Medium, brown |
| 40 | Oil Palm | HO2 | Dermaptera | Dermaptera | 3 | juvenile |
| 41 | Oil Palm | HO1 | Chilopoda | Geophilomorpha | 2 | Medium size |
| 42 | Oil Palm | HO1 | Chilopoda | Geophilomorpha | 2 | Medium size |
| 43 | Oil Palm | HO2 | Chilopoda | Geophilomorpha | 1 | 1 small |
| 44 | Oil Palm | HO2 | Chilopoda | Geophilomorpha | 1 | 1 medium size |
| 45 | Oil Palm | HO1 | Araneae | Hunting Araneae | 4 | 1 black small, 1 black small white legs, 1 medium black, free-hunters |
| 46 | Oil Palm | HO1 | Araneae | Hunting Araneae | 4 | 1 small + 1 mrdium brown white legs, 1 brown small, 1 black small white legs, free hunters |
| 47 | Oil Palm | HO2 | Araneae | Hunting Araneae | 1 | 1 black large |
| 48 | Oil Palm | HO2 | Araneae | Hunting Araneae | 1 | Large size, black, free hunter |
| 49 | Oil Palm | HO1 | Isopoda | Isopoda | 2 | Medium size cf. Oniscidae |
| 50 | Oil Palm | HO1 | Isopoda | Isopoda | 3 | Medium size cf. Oniscidae |
| 51 | Oil Palm | HO2 | Isopoda | Isopoda | 4 | Medium size cf. Oniscidae + 3 small |
| 52 | Oil Palm | HO1 | Diplopoda | Julidae | 3 | Medium size |
| 53 | Oil Palm | HO1 | Diplopoda | Julidae | 3 | Medium size |
| 54 | Oil Palm | HO3&4 | Collembola | Lepidocyrtini | 27 |  |
| 55 | Oil Palm | HO2 | Lepidoptera | Lepidoptera | 1 | medium, grey, stock |
| 56 | Oil Palm | HO1 | Mesostigmata | Mesostigmata | 10 | Uropodina |
| 57 | Oil Palm | HO2 | Mesostigmata | Mesostigmata | 34 | Uropodina |
| 58 | Oil Palm | HO2 | Mesostigmata | Mesostigmata | 21 | Gamasina |
| 59 | Oil Palm | HO2 | NA | Mixed sample | 5,3,2 | Pseudoscorpion+Entomobryidae+Coleoptera |
| 60 | Oil Palm | HO1 | Opiliones | Opiliones | 1 | Small size |
| 61 | Oil Palm | HO2 | Oribatida | Oribatida | 28 | Lohmanniididae, Otocepheidae, Galumnellidae, Scheloribatidae |
| 62 | Oil Palm | HO1 | Diplopoda | Polydesmidae | 2 |  |
| 63 | Oil Palm | HO1 | Diplopoda | Polydesmidae | 1 |  |
| 64 | Oil Palm | HO2 | Chilopoda | Scolopendromorpha | 3 | 2 small juvenile, 1 medium |
| 65 | Oil Palm | HO3&4 | Symphyla | Symphyla | 10 |  |
| 66 | Rubber | HR2 | Araneae | Araneae | 4 |  |
| 67 | Rubber | HR2 | Coleoptera | Carabidae - imago | 1 | Medium size, black, cf. Nebria |
| 68 | Rubber | HR2 | Lumbricina | Clitellata | 1 |  |
| 69 | Rubber | HR2 | Lumbricina | Clitellata | 1 |  |
| 70 | Rubber | HR3 | Lumbricina | Clitellata | 1 | large (piece) |
| 71 | Rubber | HR2 | Coleoptera | Coleoptera - predator larvae | 4 | Medium/small size, predator |
| 72 | Rubber | HR2 | Collembola | Epigeic Entomobryidae/Paronellidae | 10 | cingula |
| 73 | Rubber | HR3 | Chilopoda | Geophilomorpha | 1 | large (dead) |
| 74 | Rubber | HR2 | Diplura | Japygidae+Campodeidae | 4 |  |
| 75 | Rubber | HR1 | Collembola | Lepidocyrtini | 21 | Sinella |
| 76 | Rubber | HR2 | Collembola | Lepidocyrtini | 30 |  |
| 77 | Rubber | HR2 | Opiliones | Opiliones | 1 |  |
| 78 | Rubber | HR1&3 | Oribatida | Oribatida | 8 | Lohmanniididae |
| 79 | Rubber | HR2 | Oribatida | Oribatida | 17 | Galumnidae, Galumnellidae |
| 80 | Rubber | HR2 | Diplopoda | Polydesmidae | 2 | 1 from HR4 |
| 81 | Rubber | HR4 | Diplopoda | Polydesmidae | 1 |  |
| 82 | Rubber | HR4 | Pseudoscorpionida | Pseudoscorpionida | 22 |  |
| 83 | Rubber | HR2 | Psocoptera | Psocoptera | 10 | different ages |
| 84 | Rubber | HR2 | Schizomida | Schizomida | 1 |  |
| 85 | Rubber | HR2 | Symphyla | Symphyla | 2 |  |
| 86 | Rubber | HR2 | Collembola | Symphypleona | 7 |  |
