# Supplementary material for: Conversion of rainforest to oil palm and rubber plantations alters energy channels in soil food webs
Source: Ecol Evol. 2019 Jul 15;9(16):9027–39. doi: 10.1002/ece3.5449 (PMC6706186; doi:10.1002/ece3.5449)
Supplement: Supplementary file 2 [file ECE3-9-9027-s002.docx]

**Supplementary Table S2.** Plot-based mean values of NLFA proportions in soil fauna groups.

| **Group** | **Plot** | **System** | **2-OH 12:0** | **3-OH 12:0** | **i15:0** | **a15:0** | **2-OH 14:0** | **3-OH 14:0** | **i16:0** | **16:2 ω 6,9** | **16:3ω3,6,9** | **16:1ω7** | **16:1ω5** | **i17:0** | **cy17:0** | **2-OH 16:0** | **18:3ω6** | **18:2w6,9** | **18:1ω9, 18:3ω3** | **18:1ω7,** | **cy19:0** | **20:4ω6,9,12,15** | **20:5ω3,6,9,12,15** | **22:0** | **24:0** |
| --- | --- | --- | --- | --- | --- | --- | --- | --- | --- | --- | --- | --- | --- | --- | --- | --- | --- | --- | --- | --- | --- | --- | --- | --- | --- |
|  |  |  |  |  |  |  |  |  |  |  |  |  |  |  |  |  |  |  |  |  |  |  |  |  |  |
| Araneae | HF1&3 | F | 0 | 0.32 | 2.03 | 3.2 | 0.06 | 0.16 | 1.32 | 0.12 | 1.62 | 0.91 | 1.76 | 0.54 | 0 | 0 | 0.01 | 11.02 | 21.35 | 1.36 | 0.43 | 4.49 | 1.92 | 1.26 | 0.16 |
| Araneae | HF4 | F | 0.05 | 0.07 | 0.94 | 0.39 | 0.02 | 0.17 | 1.36 | 0.43 | 0.72 | 0.56 | 1.44 | 0.18 | 0 | 0.18 | 0.29 | 17.96 | 22.87 | 2.09 | 0.07 | 6.25 | 2.14 | 0 | 0 |
| Araneae | HO1 | O | 0.03±0.01 (2) | 0.03±0.04 (2) | 0.22±0.08 (2) | 0.3±0.16 (2) | 0.02±0.02 (2) | 0±0.01 (2) | 0.1±0.06 (2) | 0.26±0.19 (2) | 0.66±0.8 (2) | 8.03±9.04 (2) | 0.46±0.11 (2) | 0.16±0.23 (2) | 0.13±0.03 (2) | 0.11±0.08 (2) | 0.05±0.03 (2) | 17.45±1.86 (2) | 23.65±3.2 (2) | 0.72±0.24 (2) | 0.64±0.52 (2) | 3.64±1.34 (2) | 1.9±1.39 (2) | 0.22±0.28 (2) | 0±0.01 (2) |
| Araneae | HO2 | O | 0.7±1.2 (3) | 0.56±0.93 (3) | 2.35±1.65 (3) | 2.65±1.28 (3) | 0.06±0.04 (3) | 0.02±0.03 (3) | 0.39±0.44 (3) | 0.27±0.3 (3) | 0.26±0.22 (3) | 2.58±2.05 (3) | 0.8±0.53 (3) | 0.62±0.65 (3) | 0.35±0.33 (3) | 0.05±0.06 (3) | 0.19±0.28 (3) | 20.35±6.29 (3) | 23.69±7.81 (3) | 1.1±0.89 (3) | 1.33±1.15 (3) | 3.97±0.86 (3) | 1.62±0.73 (3) | 0.04±0.04 (3) | 0.02±0.04 (3) |
| Araneae | HR2 | R | 0 | 0.17 | 4.34 | 3.15 | 0.1 | 0.22 | 0.41 | 0.06 | 0 | 0.11 | 0 | 2.91 | 0.22 | 0.34 | 0.09 | 11.42 | 10.87 | 2.13 | 2.51 | 2.07 | 0.03 | 0.18 | 0 |
| Blattodea | HF2 | F | 0 | 0.01 | 0.91 | 0.2 | 0.01 | 0.02 | 0.74 | 0.09 | 0.32 | 1.16 | 1.42 | 0.83 | 0.69 | 0.03 | 0.35 | 34.85 | 21.79 | 2.27 | 0.13 | 2.21 | 0.02 | 0 | 0 |
| Blattodea | HO1 | O | 0.02 | 0.04 | 0.82 | 0.64 | 0.02 | 0.01 | 0.2 | 0.4 | 0.11 | 5.23 | 0.87 | 0.04 | 0.31 | 0.03 | 0.09 | 3.82 | 10.84 | 1.48 | 0.34 | 1.01 | 0.11 | 0.04 | 0 |
| Blattodea | HO2 | O | 0.02 | 0.02 | 0.44 | 0.6 | 0.01 | 0 | 0 | 0.2 | 0.38 | 5.01 | 0.01 | 0.3 | 0.19 | 0.02 | 0 | 13.88 | 33.22 | 0.4 | 0.17 | 0.23 | 0 | 0.01 | 0 |
| Blattodea | HO3&4 | O | 0.03 | 0.06 | 0.35 | 0.22 | 0.04 | 0 | 0.09 | 0.28 | 0.26 | 5.85 | 5.32 | 0.17 | 0 | 0.05 | 0.17 | 14.08 | 28.95 | 9.12 | 0.09 | 1.3 | 0.02 | 0 | 0 |
| Chilopoda | HF2 | F | 0.09±0.15 (3) | 0.28±0.16 (3) | 1.74±1.21 (3) | 1.28±1.36 (3) | 0.02±0.03 (3) | 0.02±0.03 (3) | 0.67±0.22 (3) | 0.1±0.06 (3) | 0.52±0.33 (3) | 1.18±1.02 (3) | 0.67±0.37 (3) | 0.61±0.39 (3) | 0.35±0.33 (3) | 0.07±0.1 (3) | 0.31±0.21 (3) | 13.78±2.25 (3) | 24.55±7.99 (3) | 0.94±0.75 (3) | 0.33±0.38 (3) | 2.38±2.69 (3) | 0.88±0.52 (3) | 0.39±0.6 (3) | 0±0 (3) |
| Chilopoda | HO1 | O | 0.6±0.57 (2) | 0.81±1.06 (2) | 1.5±0.94 (2) | 1.43±0.52 (2) | 0.01±0 (2) | 0±0 (2) | 0.12±0 (2) | 0.39±0.07 (2) | 0.97±0.08 (2) | 5.87±3.26 (2) | 0.43±0.18 (2) | 0.45±0.2 (2) | 0.52±0.02 (2) | 0.02±0.03 (2) | 0.17±0.11 (2) | 12.15±3.96 (2) | 26.62±4.75 (2) | 0.75±0.44 (2) | 0.3±0.37 (2) | 3.68±0.35 (2) | 1.15±0.44 (2) | 0.22±0.11 (2) | 0±0 (2) |
| Chilopoda | HO2 | O | 0.11±0.08 (3) | 0.14±0.16 (3) | 1.39±1.59 (3) | 2.05±1.78 (3) | 0.04±0.03 (3) | 0.12±0.17 (3) | 0.4±0.28 (3) | 0.08±0.1 (3) | 0.9±1.13 (3) | 2.91±2.55 (3) | 0.46±0.16 (3) | 0.61±0.4 (3) | 0.5±0.66 (3) | 0.05±0.08 (3) | 0.17±0.08 (3) | 12.6±2.86 (3) | 24.02±10.93 (3) | 1.46±0.62 (3) | 0.23±0.38 (3) | 3.75±2.75 (3) | 0.55±0.78 (3) | 0.07±0.06 (3) | 0.01±0.02 (3) |
| Chilopoda | HR3 | R | 0.74 | 1.03 | 1.7 | 0.84 | 0.01 | 0.01 | 0.81 | 0.24 | 0.74 | 1.57 | 0.48 | 0.08 | 0.22 | 0.22 | 0.4 | 7.5 | 24.48 | 1.37 | 0 | 7.14 | 2.29 | 0 | 0 |
| Coleoptera | HF2 | F | 0.04 | 0.13 | 0.33 | 1.19 | 0.01 | 0 | 1.15 | 0.03 | 0.13 | 3.22 | 0.55 | 1.93 | 0.87 | 0.08 | 0.19 | 5.35 | 28.75 | 1.49 | 0.06 | 3.49 | 0.45 | 0.25 | 0.02 |
| Coleoptera | HF4 | F | 0.02 | 0.02 | 1.99 | 0.23 | 0 | 0 | 1.83 | 0.16 | 0.64 | 1.63 | 0.81 | 2.27 | 0.29 | 0.09 | 0.34 | 8.06 | 26.3 | 1.95 | 0.05 | 4.32 | 0.36 | 0 | 0 |
| Coleoptera | HR2 | R | 0.38±0.53 (2) | 0.41±0.51 (2) | 0.66±0.93 (2) | 1.48±0.2 (2) | 0.07±0.03 (2) | 0.1±0.11 (2) | 0.44±0.01 (2) | 0.08±0.11 (2) | 4.36±5.19 (2) | 5.86±4.06 (2) | 1.77±1.17 (2) | 1.27±1.8 (2) | 0.47±0.66 (2) | 0.8±1.12 (2) | 0.12±0.17 (2) | 18.08±6.31 (2) | 17.77±4.91 (2) | 0.12±0.01 (2) | 0.82±1.16 (2) | 2.8±2.95 (2) | 0.26±0.37 (2) | 0±0 (2) | 0±0.01 (2) |
| Collembola | HF2 | F | 1.09±1.68 (3) | 1.68±1.01 (3) | 5.33±1.41 (3) | 9.02±2.98 (3) | 0.12±0.09 (3) | 0.01±0.02 (3) | 0.35±0.3 (3) | 0.06±0.1 (3) | 3.36±1.36 (3) | 0±0 (3) | 1.03±1.54 (3) | 0.94±0.61 (3) | 1.56±1.47 (3) | 0.36±0.26 (3) | 0±0.01 (3) | 2.81±1.54 (3) | 4.19±4.88 (3) | 0.08±0.14 (3) | 0.26±0.23 (3) | 0.4±0.63 (3) | 0.42±0.63 (3) | 0.91±0.68 (3) | 0.08±0.12 (3) |
| Collembola | HF4 | F | 0.5 | 0.21 | 3.64 | 4.8 | 0.05 | 0.04 | 0.45 | 0.67 | 5.66 | 0 | 0.1 | 1.92 | 0.7 | 0.05 | 0.07 | 5.88 | 11.1 | 0.6 | 0.07 | 2 | 0.04 | 0.96 | 0.04 |
| Collembola | HO3&4 | O | 0 | 0.15 | 4.9 | 2.85 | 0.12 | 0 | 0.21 | 0.15 | 6.02 | 0.4 | 0.29 | 0.07 | 0.18 | 0.03 | 0.08 | 2.87 | 17.8 | 0.14 | 0.68 | 1.76 | 1.44 | 0 | 0.16 |
| Collembola | HR1 | R | 0.05 | 0.68 | 3.34 | 5.66 | 0.02 | 0.39 | 0.62 | 0 | 5.78 | 0 | 1.99 | 1.7 | 4.38 | 0.03 | 0 | 2.85 | 10.15 | 0.24 | 0.45 | 0.17 | 0 | 0.89 | 0.14 |
| Collembola | HR2 | R | 0.2±0.27 (3) | 0.17±0.19 (3) | 2.26±2.54 (3) | 1.06±0.95 (3) | 0.04±0.04 (3) | 0.08±0.04 (3) | 0.54±0.39 (3) | 0.09±0.15 (3) | 6.45±0.41 (3) | 4.63±3.83 (3) | 4.17±5.05 (3) | 0.2±0.18 (3) | 0.54±0.79 (3) | 0.65±0.4 (3) | 0.02±0.03 (3) | 7.14±4.08 (3) | 15.18±0.65 (3) | 0.74±0.77 (3) | 0.76±1 (3) | 1.78±1.49 (3) | 0.61±1.04 (3) | 0.15±0.25 (3) | 0±0 (3) |
| Dermaptera | HO1 | O | 0±0 (2) | 0.02±0.01 (2) | 0.17±0.08 (2) | 0.18±0.08 (2) | 0.02±0.01 (2) | 0.01±0 (2) | 0.04±0.06 (2) | 0.1±0.02 (2) | 0.31±0.04 (2) | 11.61±7.75 (2) | 0.27±0.35 (2) | 0.08±0.01 (2) | 0.02±0.01 (2) | 0.01±0.01 (2) | 0.02±0 (2) | 4.61±0.6 (2) | 15.23±0.08 (2) | 20.14±27.59 (2) | 0.09±0.04 (2) | 0.31±0.33 (2) | 0.01±0.01 (2) | 0.03±0.01 (2) | 0±0 (2) |
| Dermaptera | HO2 | O | 0.03 | 0.05 | 0.9 | 1.07 | 0.01 | 0 | 0.06 | 0.26 | 0.26 | 8.01 | 0.22 | 0.28 | 0.43 | 0 | 0.09 | 7.67 | 29.34 | 0.91 | 0.13 | 0.25 | 0.29 | 0 | 0 |
| Diplopoda | HF1&3 | F | 0.03 | 0.86 | 3.37 | 5.83 | 0 | 0.07 | 0.24 | 0 | 1.66 | 0 | 2.38 | 1.23 | 4.61 | 0 | 0 | 5.35 | 6.95 | 3.35 | 0.68 | 0.65 | 0 | 0.89 | 0.22 |
| Diplopoda | HF2 | F | 0.02±0 (2) | 0.2±0.01 (2) | 1.8±0.47 (2) | 1.61±0.91 (2) | 0.06±0.02 (2) | 0.04±0.04 (2) | 0.89±0.03 (2) | 0.62±0.16 (2) | 0.71±0.37 (2) | 1.94±2.41 (2) | 1.4±0.66 (2) | 1.14±0.22 (2) | 0.3±0.12 (2) | 0.08±0.08 (2) | 0.17±0.1 (2) | 14.5±3.3 (2) | 19.69±9.03 (2) | 4.48±1.75 (2) | 1.13±1.17 (2) | 1.53±0.54 (2) | 0±0 (2) | 0.92±0.85 (2) | 1.94±2.55 (2) |
| Diplopoda | HO1 | O | 0.02±0.03 (4) | 0.02±0.01 (4) | 0.79±0.14 (4) | 0.63±0.25 (4) | 0.01±0 (4) | 0±0.01 (4) | 0.21±0.02 (4) | 0.16±0.06 (4) | 0.25±0.12 (4) | 3.42±1.76 (4) | 0.62±0.28 (4) | 0.52±0.26 (4) | 0.2±0.14 (4) | 0.02±0.02 (4) | 0.13±0.02 (4) | 10.28±1.86 (4) | 27.58±5.3 (4) | 0.91±0.96 (4) | 0.12±0.08 (4) | 1.14±0.53 (4) | 0.23±0.2 (4) | 0.1±0.11 (4) | 0.02±0.02 (4) |
| Diplopoda | HR2 | R | 0.81 | 1.08 | 1.68 | 1.67 | 0.02 | 0.06 | 0.84 | 0.15 | 3.8 | 0.07 | 0.28 | 0.57 | 0.73 | 0.05 | 0.33 | 14.3 | 27.98 | 3.21 | 0.58 | 1 | 0.59 | 0.04 | 0.03 |
| Diplopoda | HR4 | R | 0.03 | 0.2 | 8.9 | 0.34 | 0.05 | 0.04 | 0.36 | 0.18 | 2.56 | 0.02 | 0.26 | 0 | 0.27 | 0.5 | 12.66 | 13.59 | 10.36 | 0.11 | 0 | 1.86 | 0.02 | 0.05 | 0 |
| Isopoda | HF4 | F | 1.82 | 0.67 | 3.88 | 7.53 | 0.04 | 0 | 0.32 | 1.01 | 2.99 | 0 | 1.93 | 0.73 | 4.9 | 0.53 | 0.02 | 4.76 | 5.27 | 0.49 | 1.95 | 0.57 | 0.3 | 0 | 0.2 |
| Isopoda | HO1 | O | 0.01±0 (2) | 0.08±0.08 (2) | 1.18±0.43 (2) | 0.4±0.17 (2) | 0.02±0.01 (2) | 0±0 (2) | 0.66±0.09 (2) | 0.28±0.06 (2) | 0.3±0.01 (2) | 6.84±0.71 (2) | 1.35±0.01 (2) | 0.62±0.33 (2) | 0.26±0.09 (2) | 0.06±0.08 (2) | 0.15±0.03 (2) | 12.98±0.74 (2) | 21.03±2.74 (2) | 2.56±0.62 (2) | 0.4±0.46 (2) | 2.29±0.37 (2) | 0.46±0.15 (2) | 0.15±0.12 (2) | 0±0 (2) |
| Isopoda | HO2 | O | 1.07 | 0.37 | 4.74 | 4.76 | 0.03 | 0 | 0.34 | 1.18 | 0.84 | 2.02 | 0.63 | 3.02 | 2.4 | 0.53 | 0.12 | 3.95 | 10.41 | 0.21 | 2.91 | 0 | 0 | 1.05 | 0 |
| Lepidoptera | HO2 | O | 0.02 | 0.01 | 0.75 | 0.4 | 0.01 | 0.02 | 0 | 0.16 | 1.16 | 3.49 | 0.07 | 0.04 | 0.07 | 0.01 | 0 | 9.93 | 40.64 | 0.05 | 0.13 | 0.11 | 0 | 0.01 | 0 |
| Lumbricina | HF1&3 | F | 0.03±0.01 (3) | 0.36±0.24 (3) | 2.5±1.04 (3) | 2.55±1.07 (3) | 0.01±0.02 (3) | 0.23±0.24 (3) | 1.57±1.11 (3) | 0.02±0.04 (3) | 0.72±0.65 (3) | 0±0 (3) | 1.03±0.62 (3) | 1.18±1.02 (3) | 0.67±0.94 (3) | 0.59±0.52 (3) | 0.19±0.28 (3) | 13.55±3.21 (3) | 4.34±1.25 (3) | 4.59±1.57 (3) | 1.4±1.75 (3) | 16±6.9 (3) | 3.69±2.46 (3) | 0.83±0.44 (3) | 0.04±0.04 (3) |
| Lumbricina | HO3&4 | O | 0.3 | 0.09 | 2.43 | 1.6 | 0.02 | 0.06 | 1.9 | 0.31 | 1.81 | 0 | 1.34 | 0.28 | 0 | 0.45 | 0.07 | 12.85 | 6.68 | 0.09 | 3.06 | 14.69 | 2.75 | 0.01 | 0.02 |
| Lumbricina | HR2 | R | 0.09±0.07 (2) | 0.01±0.01 (2) | 2.78±0.08 (2) | 2.02±2.12 (2) | 0±0.01 (2) | 0.06±0.04 (2) | 1.54±0.31 (2) | 0.11±0.01 (2) | 0.84±1.19 (2) | 0.68±0.96 (2) | 1.23±0.56 (2) | 0.37±0.42 (2) | 0.22±0.11 (2) | 0.28±0.39 (2) | 0.14±0.18 (2) | 15.43±1.8 (2) | 6.87±0.3 (2) | 3.68±0.3 (2) | 0.2±0.28 (2) | 21.51±5.11 (2) | 5.59±1.68 (2) | 0.02±0.04 (2) | 0.08±0.06 (2) |
| Lumbricina | HR3 | R | 0.9 | 0.84 | 3.78 | 1.04 | 0.03 | 0.16 | 1.76 | 0.13 | 0 | 0.17 | 0.79 | 1.54 | 0.31 | 0.35 | 0.18 | 13.37 | 5.34 | 4.13 | 0.56 | 20.3 | 4.55 | 0.25 | 0 |
| Mesostigmata | HF2 | F | 0.59 | 0.67 | 3.24 | 6.33 | 0.13 | 0 | 0.45 | 0 | 2.93 | 0 | 1.61 | 6.23 | 4.05 | 0.26 | 0.05 | 9.25 | 13.87 | 0.89 | 0 | 2.03 | 0.64 | 0.05 | 0.17 |
| Mesostigmata | HO1 | O | 0.09 | 0.11 | 4.29 | 2.51 | 0.03 | 0 | 0.28 | 0 | 2.12 | 1.05 | 0.6 | 0 | 0.16 | 0.13 | 0.01 | 7.62 | 26.11 | 0.83 | 2.02 | 1.66 | 0 | 0 | 0 |
| Mesostigmata | HO2 | O | 1.5±2.09 (2) | 2.8±3.27 (2) | 4.67±5.93 (2) | 2.83±3.11 (2) | 0.29±0.4 (2) | 0.08±0.08 (2) | 0.35±0.01 (2) | 0.4±0.49 (2) | 2.38±0.79 (2) | 1.18±0.35 (2) | 0.48±0.18 (2) | 0.9±1.27 (2) | 0.67±0.16 (2) | 0.2±0.23 (2) | 0.02±0.02 (2) | 6.7±3.74 (2) | 16.76±13.82 (2) | 1.03±0.1 (2) | 0.41±0.44 (2) | 2.19±1.48 (2) | 0.42±0.13 (2) | 0.72±0.9 (2) | 0±0 (2) |
| Opiliones | HF4 | F | 0.21 | 0.15 | 3.92 | 8.81 | 0.07 | 0.02 | 0.32 | 0.01 | 0 | 0 | 0.46 | 3.12 | 0.97 | 0.1 | 0.01 | 4.96 | 10.92 | 0.82 | 1.32 | 1.98 | 0.2 | 0.31 | 0.02 |
| Opiliones | HO1 | O | 3.22 | 3.93 | 7.55 | 6.53 | 0.05 | 0 | 0.16 | 0.98 | 1.13 | 0 | 0.05 | 1.77 | 2.91 | 0.36 | 0.02 | 4.64 | 6.01 | 0.18 | 2.06 | 1.49 | 1.13 | 1.27 | 0.12 |
| Opiliones | HR2 | R | 0.01 | 0.1 | 4.73 | 2.87 | 0.05 | 0.16 | 0.7 | 0.18 | 0.17 | 0.86 | 0 | 0.44 | 0 | 0.02 | 0.15 | 11.87 | 13.54 | 2.67 | 0 | 4.96 | 0.08 | 0.07 | 0 |
| Oribatida | HF2 | F | 0.07 | 0.69 | 3.4 | 6.72 | 0.1 | 0 | 0.96 | 0.47 | 3.43 | 0 | 0.82 | 1 | 0.32 | 0.6 | 0 | 15.65 | 8.53 | 0 | 0.43 | 0.01 | 1.08 | 0.21 | 0.13 |
| Oribatida | HF4 | F | 3.71 | 3.55 | 5.47 | 7.45 | 0.03 | 0.01 | 0.21 | 0.93 | 2.48 | 0 | 1.4 | 0.15 | 0.04 | 0.3 | 0.01 | 4.6 | 5.05 | 0 | 3.31 | 0 | 0.35 | 0.54 | 0.14 |
| Oribatida | HO2 | O | 0 | 0.37 | 0.15 | 4.97 | 0.16 | 0.21 | 0.11 | 0.23 | 4.57 | 1.93 | 1.9 | 0 | 0 | 0.09 | 0.01 | 7.32 | 15.81 | 0 | 0.12 | 0.97 | 0 | 0.01 | 0.04 |
| Oribatida | HR1&3 | R | 0 | 0.14 | 1.51 | 2.58 | 0.12 | 0.2 | 0.18 | 0.13 | 2.69 | 0.06 | 0.17 | 0.69 | 0 | 0 | 0.36 | 1.86 | 8.13 | 0.43 | 0.13 | 1.13 | 0 | 0.03 | 10.89 |
| Oribatida | HR2 | R | 0.21 | 0.25 | 0.98 | 0.87 | 0.05 | 0.1 | 0 | 0.4 | 5.75 | 6.13 | 0.28 | 0.87 | 0 | 0.35 | 0 | 15.72 | 26.59 | 0.16 | 0.02 | 0 | 0 | 0 | 0 |
| Pseudoscorpionida | HF2 | F | 0.17 | 0.09 | 2.99 | 3.86 | 0.04 | 0 | 0.48 | 0.18 | 1.95 | 1.07 | 0.74 | 0.24 | 0.77 | 0.52 | 0.24 | 8.11 | 16.29 | 2.17 | 0.81 | 4.48 | 1.34 | 0 | 0.11 |
| Pseudoscorpionida | HR4 | R | 1.39 | 1.07 | 2.68 | 1.38 | 0.02 | 0.04 | 1.14 | 0.11 | 0.99 | 2.19 | 0.35 | 0.07 | 0.01 | 0.11 | 0.37 | 11.11 | 23.82 | 2.18 | 0 | 6 | 1.91 | 0.5 | 0 |
| Psocoptera | HR2 | R | 3.26 | 1.82 | 1.14 | 1.32 | 0 | 0 | 0.27 | 0.61 | 0.72 | 0.97 | 0.31 | 0.57 | 0.48 | 0 | 0.33 | 20.59 | 16.88 | 0.97 | 0.31 | 0.13 | 0 | 0.46 | 0 |
| Schizomida | HF4 | F | 0.05 | 0.16 | 1.48 | 7.01 | 0 | 0.63 | 0 | 0 | 2.21 | 0 | 3.11 | 4.73 | 3.72 | 0 | 0 | 10.41 | 13.57 | 0.79 | 1.52 | 9.39 | 1.98 | 0.11 | 0.02 |
| Schizomida | HR2 | R | 0.33 | 0.15 | 4.55 | 2.94 | 0.03 | 0 | 0.62 | 0.27 | 0 | 1.49 | 0.42 | 0 | 0.19 | 0.05 | 0.2 | 12.52 | 14.04 | 0.54 | 0 | 0.12 | 1.7 | 0.27 | 0 |
| Symphyla | HF1&3 | F | 0.01 | 0.01 | 0.02 | 1.48 | 0.03 | 0.04 | 3.03 | 0.16 | 4.97 | 5.58 | 0.83 | 1.18 | 1.18 | 0.13 | 0.12 | 7.11 | 27.03 | 0.77 | 0.23 | 2.12 | 0.08 | 0.5 | 0.19 |
| Symphyla | HO3&4 | O | 0 | 0.07 | 4.75 | 2.49 | 0 | 0.06 | 0.25 | 0.16 | 0.71 | 2.63 | 0.25 | 0 | 0.52 | 0.07 | 0.13 | 3.37 | 21.63 | 1.92 | 0 | 0.04 | 0 | 0.01 | 0.05 |
| Symphyla | HR2 | R | 4.01 | 6.78 | 9.61 | 5.75 | 0.06 | 0.01 | 0.43 | 0.26 | 0.98 | 0.05 | 0 | 0 | 0 | 0.35 | 0.02 | 2.96 | 5.28 | 0.18 | 2.07 | 0.41 | 0.01 | 0.13 | 0 |
