# Supplementary material for: Conversion of rainforest to oil palm and rubber plantations alters energy channels in soil food webs
Source: Ecol Evol. 2019 Jul 15;9(16):9027–39. doi: 10.1002/ece3.5449 (PMC6706186; doi:10.1002/ece3.5449)
Supplement: Supplementary file 3 [file ECE3-9-9027-s003.docx]

**Table S3.** Average proportions of biomarker FAs in dominating taxa of soil animals.

| Biomarker, NLFA | Araneae | | Chilopoda | Collembola | Diplopoda | Lumbricina | Oribatida |  |
| --- | --- | --- | --- | --- | --- | --- | --- | --- |
| **Algae** |  | |  |  |  |  |  | |
| 16:2ω6,9 | 0.23 ± 0.14 (5) | | 0.2 ± 0.14 (4) | 0.19 ± 0.27 (5) | 0.22 ± 0.24 (5) | 0.14 ± 0.12 (4) | 0.43 ± 0.31 (5) | |
| 16:3ω3,6,9 | 0.65 ± 0.62 (5)c | | 0.78 ± 0.2 (4)c | 5.46 ± 1.21 (5)a | 1.8 ± 1.43 (5)bc | 0.84 ± 0.74 (4)c | 3.78 ± 1.37 (5)ab | |
| AMF |  | |  |  |  |  |  | |
| 16:1ω5 | 0.89 ± 0.72 (5) | | 0.51 ± 0.11 (4) | 1.51 ± 1.66 (5) | 0.99 ± 0.91 (5) | 1.1 ± 0.24 (4) | 0.91 ± 0.74 (5) | |
| **Animal-synthesized ^*^** |  | |  |  |  |  |  | |
| 20:4ω6 | 4.08 ± 1.51 (5)b | | 4.24 ± 2.03 (4)b | 1.22 ± 0.86 (5)c | 1.24 ± 0.47 (5)c | 18.13 ± 3.29 (4)a | 0.42 ± 0.58 (5)c | |
| 20:5ω3 | 1.52 ±0. 85 (5)b | | 1.22 ± 0.76 (4)bc | 0.5 ± 0.58 (5)bc | 0.17 ± 0.25 (5)c | 4.15 ± 1.21 (4)a | 0.29 ± 0.47 (5)c | |
| **Fungi** |  | |  |  |  |  |  | |
| 18:2ω6,9 | 15.64 ± 4.18 (5)a | | 11.51 ± 2.76 (4)a | 4.31 ± 2.06 (5)b | 11.6 ± 3.89 (5)a | 13.8 ± 1.13 (4)a | 9.03 ± 6.37 (5)ab | |
| **Non-specific bacteria** | |  |  |  |  |  |  | |
| 18:1ω7  **Gram-negative bacteria** | 1.48 ± 0.62 (5)ab | | 1.13 ± 0.34 (4)ab | 0.36 ± 0.29 (5)bc | 2.41 ± 1.83 (5)a | 3.12 ± 2.06 (4)a | 0.12 ± 0.19 (5)c | |
| 2-OH 12:0 | 0.16 ± 0.3 (5) | | 0.38 ± 0.33 (4) | 0.37 ± 0.45 (5) | 0.18 ± 0.35 (5) | 0.33 ± 0.4 (4) | 0.8 ± 1.63 (5) | |
| 3-OH 12:0 | 0.23 ± 0.21 (5) | | 0.56 ± 0.42 (4) | 0.58 ± 0.65 (5) | 0.47 ±0 .47 (5) | 0.32 ± 0.37 (4) | 1 ± 1.44 (5) |  |
| 2-OH 14:0 | 0.05 ± 0.03 (5)ab | | 0.02 ± 0.01 (4)b | 0.07 ± 0.05 (5)ab | 0.03 ± 0.02 (5)b | 0.02 ± 0.01 (4)b | 0.09 ± 0.05 (5)a | |
| 3-OH 14:0 | 0.12 ± 0.1 (5) | | 0.04 ± 0.06 (4) | 0.1 ± 0.16 (5) | 0.04 ± 0.02 (5) | 0.13 ± 0.08 (4) | 0.1 ± 0.1 (5) |  |
| cy17:0 | 0.14 ± 0.15 (5)ab | | 0.4 ± 0.14 (4)ab | 1.47 ± 1.7 (5)a | 1.22 ± 1.91 (5)ab | 0.3 ± 0.28 (4)ab | 0.07 ± 0.14 (5)b | |
| 2-OH 16:0 | 0.13 ± 0.13 (5) | | 0.09 ± 0.09 (4) | 0.22 ± 0.27 (5) | 0.13 ± 0.21 (5) | 0.42 ± 0.14 (4) | 0.27 ± 0.24 (5) | |
| cy19:0  **Gram-positive bacteria** | 1 ± 0.96 (5) | | 0.21 ± 0.15 (4) | 0.44 ± 0.29 (5) | 0.5 ± 0.46 (5) | 1.3 ± 1.27 (4) | 0.8 ± 1.41 (5) | |
| i15:0 | 1.98 ± 1.57 (5) | | 1.58 ± 0.16 (4) | 3.89 ± 1.24 (5) | 3.31 ± 3.26 (5) | 2.87 ± 0.62 (4) | 2.3 ± 2.14 (5) | |
| a15:0 | 1.94 ± 1.47 (5) | | 1.4 ± 0.5 (4) | 4.68 ± 3.01 (5) | 2.02 ± 2.21 (5) | 1.8 ± 0.64 (4) | 4.52 ± 2.77 (5) | |
| i16:0 | 0.72 ± 0.58 (5)b | | 0.5 ± 0.3 (4)b | 0.43 ± 0.16 (5)b | 0.51 ± 0.33 (5)b | 1.69 ± 0.17 (4)a | 0.29 ± 0.38 (5)b | |
| i17:0  **Plants** | 0.88 ± 1.15 (5) | | 0.44 ± 0.25 (4) | 0.97 ± 0.84 (5) | 0.69 ± 0.5 (5) | 0.84 ± 0.62 (4) | 0.54 ± 0.44 (5) | |
| 18:3ω6,9,12 | 0.13 ± 0.11 (5) | | 0.26 ± 0.11 (4) | 0.03 ± 0.04 (5) | 2.66 ± 5.59 (5) | 0.14 ± 0.06 (4) | 0.08 ± 0.16 (5) | |
| 18:1ω9, | 20.49±5.46 (5)ab | | 24.92 ± 1.16 (4)a | 11.68 ±5.21 (5)bc | 18.51±9.66 (5)ab | 5.81 ± 1.19 (4)c | 12.82 ± 8.65 (5)ac | |
| 22:0 | 0.34 ± 0.52 (5) | | 0.17 ± 0.17 (4) | 0.58 ± 0.47 (5) | 0.4 ± 0.46 (5) | 0.28 ± 0.38 (4) | 0.16 ± 0.23 (5) | |
| 24:0 | 0.04 ± 0.07 (5) | | 0 ± 0 (4) | 0.08 ± 0.07 (5) | 0.44 ± 0.84 (5) | 0.03 ± 0.03 (4) | 2.24 ± 4.84 (5) | |

^*^Animal-synthesized C20 polyunsaturated FAs cannot be used as biomarkers for animal consumption

**Table S4.** LDA coefficients for each of the analyzed biomarker FA indices.

| Biomarker NLFA | LD1 | LD2 |
| --- | --- | --- |
| Algae | 0.2426243 | -10.149924 |
| AMF | -10.1487733 | 19.781153 |
| Animal-synthesized^*^ | -1.1068080 | -1.691835 |
| Non-spesific bacteria | 3.6290698 | -2.243505 |
| Spesific bacteria (sum) | -5.9228615 | 2.621853 |
| Fungi | -4.3352754 | -6.181481 |
| Plants | 0.5622746 | 7.185056 |

^*^Animal-synthesized C20 polyunsaturated FAs cannot be used as biomarkers for animal consumption

**Table S5.** Biomarker FAs composition in soil animals from different land-use systems. Means and standard deviations across all taxa are shown (number of replicates is given in brackets). Significant differences from forest based on linear mixed-effects models are shown in bold; *p < 0.05; **p < 0.01; ***p < 0.001.

| **Biomarker** | **FA** | **Rainforest** | **Rubber plantation** | **Oil palm plantation** |
| --- | --- | --- | --- | --- |
| AMF | 16:1ω5 | 1.24 ± 0.72 (19) | **0.81 ± 1.12 (15)*** | 0.93 ± 1.23 (17) |
| Algae | 16:2ω6,9 | 0.27 ± 0.32 (19) | 0.16 ± 0.1 (15) | 0.34 ± 0.3 (17) |
| Algae | 16:3ω3,6,9 | 1.95 ± 1.63 (19) | 2.34 ± 2.34 (15) | 1.39 ± 1.63 (17) |
| Animal-synthesized ^*^ | 20:4ω6,9,12,15 | 3.38 ± 3.85 (19) | 4.75 ± 6.91 (15) | 2.58 ± 3.38 (17) |
| Animal-synthesized ^*^ | 20:5ω3,6,9,12,15 | 0.84 ± 0.98 (19) | 1.18 ± 1.77 (15) | 0.69 ± 0.83 (17) |
| Fungi | 18:2ω6,9 | 10.42 ± 7.33 (19) | 10.65 ± 5.06 (15) | 9.82 ± 5.27 (17) |
| General bacteria | 16:1ω7 | 0.91 ± 1.44 (19) | 1.59 ± 2.17 (15) | **3.23 ± 2.49 (17)***** |
| General bacteria | 18:1ω7 | 1.53 ± 1.37 (19) | 1.46 ± 1.43 (15) | 1.35 ± 2.12 (17) |
| Gram^+^-bacteria | i15:0 | 2.58 ± 1.54 (19) | 3.57 ± 2.64 (15) | 2.5 ± 2.22 (17) |
| Gram^+^-bacteria | a15:0 | 4.18 ± 3.07 (19) | **2.24 ± 1.64 (15)*** | **2.2 ± 1.83 (17)*** |
| Gram^+^-bacteria | i16:0 | 0.86 ± 0.73 (19) | 0.69 ± 0.48 (15) | **0.34 ± 0.43 (17)**** |
| Gram^+^-bacteria | i17:0 | 1.59 ± 1.58 (19) | **0.71 ± 0.83 (15)*** | **0.56 ± 0.77 (17)**** |
| Gram^-^-bacteria | 2-OH 12:0 | 0.45 ± 0.92 (19) | 0.61 ± 1.03 (15) | 0.45 ± 0.84 (17) |
| Gram^-^-bacteria | 3-OH 12:0 | 0.53 ± 0.84 (19) | 0.87 ± 1.68 (15) | 0.57 ± 1.09 (17) |
| Gram^-^-bacteria | 2-OH 14:0 | 0.04 ± 0.04 (19) | 0.05 ± 0.03 (15) | 0.05 ± 0.07 (17) |
| Gram^-^-bacteria | 3-OH 14:0 | 0.08 ± 0.15 (19) | 0.11 ± 0.1 (15) | 0.03 ± 0.06 (17) |
| Gram^-^-bacteria | cy17:0 | 1.37 ± 1.63 (19) | **0.5 ± 1.09 (15)*** | **0.55 ± 0.82 (17)*** |
| Gram^-^-bacteria | 2-OH 16:0 | 0.21 ± 0.21 (19) | 0.27 ± 0.24 (15) | 0.13 ± 0.16 (17) |
| Gram^-^-bacteria | cy19:0 | 0.75 ± 0.86 (19) | 0.54 ± 0.77 (15) | 0.87 ± 1.01 (17) |
| Plants | 18:3ω6,9,12 | 0.13 ± 0.13 (19) | 1 ± 3.23 (15) | 0.09 ± 0.07 (17) |
| Plants | 18:1ω9 | 15.39 ± 8.46 (19) | 14.69 ± 7.76 (15) | **20.05 ± 7.99 (17)*** |
| Plants | 22:0 | 0.43 ± 0.41 (19) | 0.17 ± 0.24 (15) | 0.23 ± 0.39 (17) |
| Plants | 24:0 | 0.18 ± 0.43 (19) | 0.74 ± 2.81 (15) | 0.03 ± 0.05 (17) |

^*^Animal-synthesized C20 polyunsaturated FAs cannot be used as biomarkers for animal consumption
